# Evaluation of Reference Genes and Expression Level of Genes Potentially Involved in the Mode of Action of Cry1Ac and Cry1F in a Susceptible Reference Strain of *Chrysodeixis includens*

**DOI:** 10.3390/insects12070598

**Published:** 2021-06-30

**Authors:** Macarena Martin, Debora Boaventura, Ralf Nauen

**Affiliations:** 1 Department of Agronomy, Food, Natural Resources, Animals and Environment, University of Padova, 35020 Padova, Italy; macamar_20@hotmail.com; 2 Bayer AG, Crop Science Division, R&D Pest Control, Alfred Nobel Str. 50, 40789 Monheim, Germany; debora.duarteboaventura@bayer.com

**Keywords:** soybean looper, *Bacillus thuringiensis*, resistance, reference genes, *ABCC2*

## Abstract

**Simple Summary:**

Soybean looper (a moth species) is a major pest of soybean plants in the American continent and its larvae need to be kept under economic damage thresholds to guarantee sustainable yields. Soybean looper control relies mostly on the use of insecticides and genetically modified crops expressing *Bacillus thuringiensis* (Bt) insecticidal proteins. Due to the high selection pressure exerted by these control measures, resistance has developed to different insecticides and Bt proteins. Here, we tested the basal sensitivity of a soybean looper laboratory reference strain against two insecticidal proteins and determined the level of expression of potential receptors of these proteins across all (six) larval stages. Furthermore, we identified stable reference genes across all larval stages to normalize gene expression data obtained by quantitative polymerase chain reaction (qPCR). The results presented in this communication are useful to support future studies on insecticide and insecticidal protein resistance in soybean looper.

**Abstract:**

Soybean looper (SBL), *Chrysodeixis includens* (Walker), is one of the major lepidopteran pests of soybean in the American continent. SBL control relies mostly on the use of insecticides and genetically modified crops expressing *Bacillus thuringiensis* (Bt) insecticidal Cry proteins. Due to the high selection pressure exerted by these control measures, resistance has developed to different insecticides and Bt proteins. Nevertheless, studies on the mechanistic background are still scarce. Here, the susceptibility of the laboratory SBL-Benzon strain to the Bt proteins Cry1Ac and Cry1F was determined in diet overlay assays and revealed a greater activity of Cry1Ac than Cry1F, thus confirming results obtained for other sensitive SBL strains. A reference gene study across larval stages with four candidate genes revealed that *RPL10* and *EF1* were the most stable genes for normalization of gene expression data obtained by RT-qPCR. Finally, the basal expression levels of eight potential Bt protein receptor genes in six larval instars were analyzed, including ATP-binding cassette (*ABC*) transporters, alkaline phosphatase, aminopeptidases, and cadherin. The results presented here provide fundamental knowledge to support future SBL resistance studies.

## 1. Introduction

Soybean looper (SBL), *Chrysodeixis includens* (Walker) (Lepidoptera: Noctuidae), is a distinctive species from the Western hemisphere and a key pest of soybean in many countries of the American continent [1]. The control of SBL relies on genetically modified crops expressing *Bacillus thuringiensis* (Bt) insecticidal proteins and foliar insecticide applications [2].

Frequent insecticide applications have led to insecticide resistance against some major chemical classes, such as pyrethroids, carbamates, organophosphates, benzoylureas, and diamides [3,4,5]. Moreover, the high selection pressure exerted by millions of hectares of soybean expressing Bt proteins has resulted in resistance towards Cry1Ac [6].

A reduction in Cry protein binding due to changes in the expression and/or mutations in midgut receptors involved in Cry protein-mediated intoxication was the most frequent mechanism of resistance [7]. Different proteins have been identified as receptors for Cry proteins in lepidopteran species, including aminopeptidases (*APN*), membrane-bound alkaline phosphatases (m*ALP*), cadherins (*CAD*), and ATP-binding cassette (*ABC*) transporters [8].

Brazilian field populations of SBL selected against Cry1Ac in the laboratory showed up to 127-fold resistance [6]. However, the molecular mechanism involved in the resistance of SBL towards Cry1Ac is still unknown. In other lepidopteran pest species, resistance to Cry1Ac has been associated with mutations and/or differences in the expression levels of *ABCC2*, *APN*, m*ALP*, and *CAD* [9,10,11,12,13].

Here, we have tested the basal sensitivity of the commonly known reference SBL-Benzon strain towards two major Bt proteins, Cry1Ac and Cry1F. Moreover, we have investigated, for the first time, the stability of potential reference genes with low expression variance across larval instars of SBL for normalization of RT-qPCR expression data. As resistance towards Bt proteins in other species has been linked to differences in the expression of potential Bt receptor genes, we have analyzed the basal expression of eight potential Bt protein receptor genes in six larval instars to support the characterization of resistance mechanisms to Cry toxins in SBL in future studies.

## 2. Material and Methods

### 2.1. Insects

SBL-Benzon strain from Benzon Research Inc., USA was reared in the laboratory under controlled conditions (25 ± 1°C, 55 ± 5% relative humidity, and a photoperiod of L16:D8). Briefly, adults were kept in cages covered with filter paper for egg laying and fed with honey water pads every second day. Larvae were mass-kept in a plastic box (18 × 13 × 5 cm) containing Soybean-Looper-Diet (Southland Products Inc., Lake Village, AR, USA) until the third instar. Afterwards, two third-instar larvae were confined to petri dishes (diameter 6 cm) containing the same diet until pupation.

### 2.2. Cry1Ac and Cry1F Bioassays

The bioassays were performed according to Boaventura et al. 2020 [14]. Briefly, seven concentrations of Cry1Ac (450-0.62 ng cm^−2^) and eight concentrations of Cry1F (12,150-5.56 ng cm^−2^) were tested. Dilutions were prepared from Cry1Ac lyophilized material (28.2% (*w*/*w*) purity) and Cry1F (TIC-842) crystal spore solution [15] in 50 mM sodium carbonate buffer (pH 10.4) and 0.1% (*v*/*v*) Triton X-100. Twenty-five μL of Bt solution was applied to the surface of artificial diet in a 48-well plate (Greiner CELLSTAR^®^, Merck, Darmstadt, Germany) and a single SBL neonate larva (<24 h old) was placed into each well and kept at 20 ± 2 °C.

A total number of 12 neonates were used per protein concentration and control (buffer and 0.1% (*v*/*v*) Triton X-100). For the highest concentration, only six neonates were used and the other six neonates were placed in wells containing only artificial diet. The bioassay was replicated three times and the mortality was scored after five days; larvae that did not move or reach the third instar were considered dead. The results were fitted by a logistic regression model to calculate LC_50_/LC_95_ values and 95% confidence intervals (GraphPad Software Inc. v.5, San Diego, CA, USA).

### 2.3. RNA Extraction and cDNA Synthesis

Total RNA from each instar was extracted from 30 pooled (first instar) or 10 pooled larvae (second to sixth instar) of SBL in four biological replicates. The RNA extraction from first and second instars was performed with the PicoPure^TM^ RNA Isolation Kit (Thermo Fisher, Vilnius, Lithuania) according to the manufacturer’s instructions. For the third to sixth instar, the larval tissue was ground with a Retsch TissueLyser (QIAGEN, Hilden, Germany) or mortar and pestle. The total RNA was extracted from powdered samples using TRIzol^®^ reagent (Invitrogen, Waltham, MA, USA) followed by RNA purification according to RNeasy^®^ Plus Universal Mini Kit (QIAGEN, Hilden, Germany) recommendations including gDNA elimination with DNase I. The RNA concentration was normalized to 100 ng μL^−1^ and 1 μg total RNA was used in 20 μL reactions for cDNA synthesis using iScript™ cDNA synthesis (Bio-Rad, Laboratories, CA, USA) according to the manufacturer’s instructions.

### 2.4. Screening for Reference Genes

The expression of four candidate reference genes was tested: *ribosomal protein L10* (*RPL10*), *glyceraldehyde-3-phosphate dehydrogenase* (*GAPDH*), *elongation factor-1 alpha* (*EF1*), and *beta actin 1* (*ACT1*). The reference gene sequences were obtained from a custom-made SBL transcriptome generated at Bayer AG through queries using local BLAST searches and submitted to GenBank (Table S1). The primers were designed using Geneious software v. 10.2.3 (Biomatters Ltd, Auckland, New Zealand) (Table S1). The RT-qPCR reactions were performed as recently described [14] using reverse/forward primers shown in Table S1. Amplification efficiencies were determined by a series of 5-fold dilutions to create standard curves by a linear regression model [16] (Table S1).

The cycle threshold values (Ct values) were obtained from CFX Maestro 1.0 v4.0 (Bio-Rad) software. The expression stability of four candidate reference genes was analyzed using geNorm qBase Plus v3.1 software (Biogazelle, Belgium) and NormFinder (moma.dk/normfinder-software) using stability value (M) below 0.5 and pairwise variation (V) value below 0.15 [16]. The Excel-based tool Normfinder was used according to Andersen et al. (2004) [17].

### 2.5. Expression Analysis of Possible Bt Protein Target Genes

The expression level of eight potential Bt receptor genes (*ABCC2*, *ABCC3*, *CAD*, m*ALP*, and *APN* (*APN1-4*)) was determined by RT-qPCR in six larval instars. The genes *EF1* and *RPL10* were the most stable genes across SBL larval instars and were used for normalization. In total, four biological replicates containing cDNA from a pool of 10-30 larvae, as described in Section 2.3, were used for analyzing the normalized expression levels. Primer pairs are described in Table S1 and the RT-qPCR conditions were chosen as recently described [14]. The normalized expression values obtained for the different developmental stages were compared to the Ct values from the first instar and statistically tested by one-way ANOVA, followed by Tukey–Kramer multiple-comparison test with qbase + software (v. 3.2; Biogazelle, Belgium).

## 3. Results

### 3.1. Bioassays

**Figure 1:**
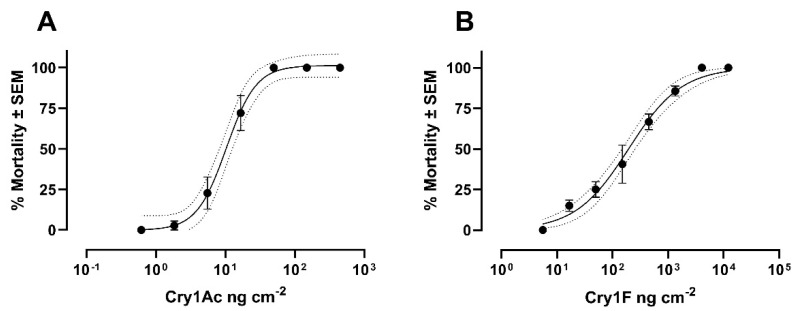
Toxicity of Bt proteins against neonates of soybean looper strain SBL-Benzon. **(A)** Toxicity of Cry1Ac (N = 288) and **(B)** toxicity of Cry1F (N = 324). Dashed lines indicate the 95% confidence band.

For Cry1Ac, the LC_50_ and LC_95_ values were 10.41 ng cm^−2^ and 44.56 ng cm^−2^, respectively. Cry1F showed an LC_50_ of 228.1 ng cm^−2^ and an LC_95_ of 4,967 ng cm^−2^ (Figure 1, Table S2).

### 3.2. Screening for Reference Genes

According to geNorm, *RPL10* was identified as the most stable gene across larval stages of SBL (M = 0.458), followed by *EF1*, *GAPDH*, and *ACT1* with M values of 0.519, 0.559, and 0.637, respectively (Table 1). When using NormFinder, *EF1* (stability value = 0.242) and *RPL10* (0.244) were the most stable genes, followed by *GAPDH* (0.309) and *ACT1* (0.351) (Table 1).

### 3.3. Expression Analysis of Potential Bt Protein Target Genes

**Figure 2:**
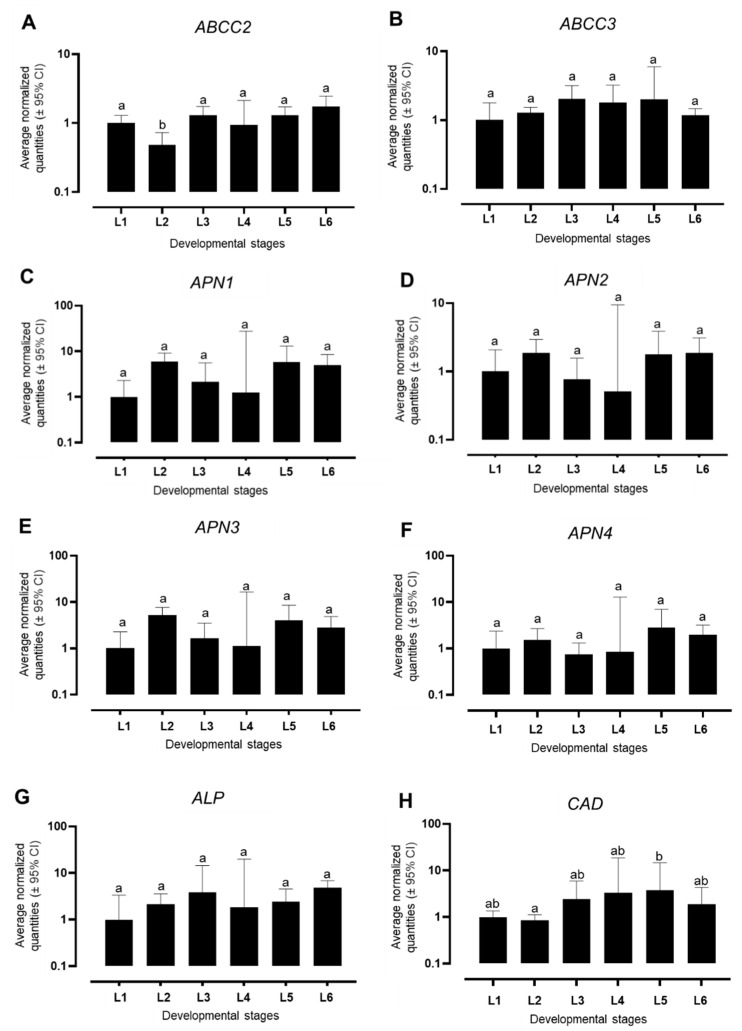
RT-qPCR expression analysis of genes possibly involved in Bt toxicity, analyzed by geNorm across different larval stages of *Chrysodeixis includens*. Average normalized quantities of (**A**) *ABCC2,* (**B**) *ABCC3*, (**C**) *APN1*, (**D**) *APN2*, (**E**) *APN3*, (**F**) *APN4*, (**G**) *ALP*, and (**H**) *CAD*. *CAD*: cadherin; *ABCC2*/*ABCC3*: ATP-binding cassette subfamily C2/C3 transporter; *ALP*: alkaline phosphatase; *APN*: amino peptidase class 1, 2, 3, and 4. Different letters denote a significant difference (One-way ANOVA; post hoc Tukey comparison, *p* < 0.05).

The basal expression levels of the genes *ABCC2*, *ABCC3*, *CAD*, m*ALP*, and *APN (APN1-4)*, reported to be potential receptors of Cry1Ac and Cry1F in other lepidopteran species, were analyzed in the different larval stages of the Bt-susceptible SBL-Benzon strain. For *ABCC3*, no significant difference in the expression level across larval stages was observed, with average normalized values between 1 and 2.02. In contrast, the expression level of *ABCC2* was significantly lower in second-instar larvae (*p*-value < 0.05) (Figure 2).

The expression level of the individual *APN* genes was not significantly different between the different larval stages. Furthermore, no significant differences in the expression level of m*ALP* were detected. However, the expression level of *CAD* in second-instar larvae was significantly lower than in fifth-instar larvae (Figure 2).

## 4. Discussion

The use of insecticide-susceptible reference strains such as SBL-Benzon is a key aspect in resistance monitoring and mechanism studies. SBL is a notorious pest of soybean crops, but it also feeds on cotton [18]. Cry1Ac is utilized in both crops and a relatively high survival rate of SBL in Cry1Ac Bt cotton has been reported [19]. The primary Insecticide Resistance Management (IRM) strategy for Bt crops has been the use of refuges (presence of non-Bt host plants near Bt crops). The success of refuge-based IRM depends on a low frequency of potential resistance alleles, high susceptibility to the expressed Bt protein(s), and a recessive mode of inheritance [20]. Utilizing Bt crops expressing more than one Bt protein (pyramid strategy), such as Bt soybean expressing Cry1Ac and Cry1F, is another valuable IRM strategy [21]. The combination of Cry1F and Cry1Ac in transgenic cotton was shown to be highly effective against SBL [22]. However, soybean and cotton are usually planted in succession in Brazil, thus increasing the resistance risk of SBL by continuous selection pressure, particularly against Cry1F, which exhibited significantly lower activity than Cry1Ac against the susceptible reference strain SBL-Benzon. The data obtained here were close to dose–response data published recently and showing the superior efficacy of Cry1Ac compared to Cry1F against neonates of a susceptible Brazilian SBL field strain [6]. This is in contrast to toxicity data previously published in another study, where Cry1F was more active than Cry1Ac against neonates of a sensitive SBL laboratory colony, whereas brush-border membrane vesicle (BBMV) binding studies revealed the opposite, i.e., a higher affinity of Cry1Ac than Cry1F [23].

The mechanisms driving Bt resistance are quite diverse and often associated with the differential expression of genes of potential Bt receptor proteins, particularly in noctuid pests reviewed in [24]. Bt protein resistance in SBL is not yet widespread, and mechanistic studies of factors driving resistance in SBL are scarce. However, the elucidation of mechanisms conferring Bt resistance based on differential gene expression analysis requires stable reference genes for normalization of RT-qPCR data [25]. Such studies have not yet been conducted in SBL, so we investigated the stability of four candidate reference genes across six larval instars to allow the selection of at least two appropriate reference genes typically considered sufficient for the normalization of RT-qPCR data to reduce any systematic bias [26]. Based on the results obtained in our study, *RPL10* and *EF1* were most stably expressed across larval stages of SBL and deemed suitable to be used in future gene expression studies. However, *EF1* was recently already employed for the normalization of the expression level of detoxification genes in a pyrethroid-resistant SBL strain [27].

Finally, we utilized the identified reference genes to normalize RT-qPCR data obtained for the basal expression level of potential Bt receptor genes in strain SBL-Benzon across six larval instars. Such data are a useful resource for the comparative analysis of gene expression levels in future studies with SBL strains resistant to Bt proteins. *ABCC2* has been described as an important receptor for Cry1Ac [10] and Cry1F [28] in lepidopteran pests, and mutations in *ABCC2* were shown to confer resistance to Cry1 proteins in *H. armigera* [10] and other noctuid pests such as fall armyworm [14,15,24]. Meanwhile, in *Plutella xylostella*, Cry1Ac resistance was linked to the downregulation of *ABCC2*, *ABCC3*, and *mALP* [13]. However, in a *Helicoverpa zea* strain selected with Cry1Ac, resistance was associated with an increase in the expression of *ALP* [29]. Target-site mutations in *APN1* and differences in its expression level have been reported to cause resistance to Cry1Ac in *H. armigera* and *Trichoplusia ni,* respectively [9,11]. Cadherins were also shown to be receptors of Cry1Ac in different lepidopteran species [24,30]. Differences in cadherin levels and/or mutations in this gene were, for example, associated with Cry1Ac resistance in *Ostrinia furnacalis* [12]. Our results reveled no differences in *APN* and *ALP* expression levels across larval instars of Bt-susceptible SBL, but significant differences in *CAD* expression levels between the second and fifth larval instar; however, additional work will be necessary to investigate if such differences have implications for Bt protein toxicity.

In summary, we have shown the stability of the genes *RPL10* and *EF1* across six different larval stages of SBL and recommend their use for the normalization of gene expression data in future studies. In addition, we identified and described potential Bt target genes in SBL and investigated their basal expression profiles across larval instars in the reference SBL-Benzon strain to support future resistance studies in SBL.

## 5. Conclusions

Resistance towards Bt proteins has already been reported in SBL and the results presented in this study will help to support future research on SBL resistance towards both Bt proteins and synthetic insecticides. Besides the data presented on the susceptibility towards Cry1Ac and Cry1F proteins, this study provides useful insights into the stability of reference genes, with *RPL10* and *EF1* as the most stable ones. Moreover, for the first time, potential Bt target genes (*ABCC2*, *ABCC3*, *cadherin*, m*ALP*, and several *APNs*) and their respective expression profiles were investigated for a susceptible SBL reference strain, SBL-Benzon.

## Figures and Tables

**Table 1:** Stability analysis of the candidate reference genes *ribosomal protein L10* (*RPL10*), *glyceraldehyde-3-phosphate dehydrogenase* (*GAPDH*), *elongation factor-1 alpha* (*EF1*), and *beta actin 1* (*ACT1*) analyzed by geNorm (average expression stability or M value) and NormFinder (stability value) in different larval stages of *Chrysodeixis includens*.

Method	*EF1*	*RPL10*	*GAPDH*	*ACT1*
geNorm	0.519	0.458	0.559	0.637
NormFinder	0.242	0.244	0.309	0.351

## Data Availability

The data presented in this study are available in the supplementary materials or on request from the corresponding author.

## Supplementary Materials

The following are available online at https://www.mdpi.com/article/10.3390/insects12070598/s1, Table S1: Gene names of orthologous candidate reference genes (*RPL10*, *GADPH*, *EF1*, and *ACT1*), potential orthologous Cry toxin target genes, species, GenBank accession numbers and primer sequences for the amplification of the respective Chrysodeixis includens genes, Table S2: Toxicity (LC50-and LC95-values) of Cry1Ac and Cry1F to neonates of soybean looper Benzon strain and the respective goodness of fit calculated for the non-linear regressions used.

## References

[1] Herzog D.C., Kogan M., Herzog D.C. (1980). Sampling Soybean Looper on Soybean. Sampling Methods in Soybean Entomology.

[2] Di Oliveira J.R.G., Ferreira M.D.C., Román R.A.A. (2010). Diferentes diâmetros de gotas e equipamentos para aplicação de inseticida no controle de Pseudoplusia includens. Eng. Agrícola.

[3] Boernel D.J., Mink J.S., Wier A.T., Thomas J.D., Leonard B.R., Gallardo F., Copping L.G., Green M.B.M., Rees R.T. (1992). Management of Insecticide Resistant Soybean Loopers (Pseudoplusia Includens) in the Southern United States. Pest Management in Soybean.

[4] Owen L.N., Catchot A., Musser F., Gore J., Cook D., Jackson R. (2013). Susceptibility ofChrysodeixis includens(Lepidoptera: Noctuidae) to Reduced-Risk Insecticides. Fla. Èntomol..

[5] Stacke R.F., Godoy D., Pretto V.E., Führ F.M., Gubiani P.D.S., Hettwer B.L., Garlet C.G., Somavilla J.C., Muraro D.S., Bernardi O. (2020). Field-evolved resistance to chitin synthesis inhibitor insecticides by soybean looper, Chrysodeixis includens (Lepidoptera: Noctuidae), in Brazil. Chemosphere.

[6] Rodrigues-Silva N., Canuto A.F., Oliveira D.F., Teixeira A.F., Amaya O.F.S., Picanço M.C., Pereira E.J.G. (2019). Negative cross-resistance between structurally different Bacillus thuringiensis toxins may favor resistance management of soybean looper in transgenic Bt cultivars. Sci. Rep..

[7] Heckel D.G., Gahan L.J., Baxter S.W., Zhao J., Shelton A.M., Gould F., Tabashnik B.E. (2007). The diversity of Bt resistance genes in species of Lepidoptera. J. Invertebr. Pathol..

[8] Bravo A., Gill S.S., Soberón M. (2007). Mode of action of Bacillus thuringiensis Cry and Cyt toxins and their potential for insect control. Toxicon.

[9] Tiewsiri K., Wang P. (2011). Differential alteration of two aminopeptidases N associated with resistance to Bacillus thuringiensis toxin Cry1Ac in cabbage looper. Proc. Natl. Acad. Sci. USA.

[10] Xiao Y., Zhang T., Liu C., Heckel D., Li X., Tabashnik B.E., Wu K. (2014). Mis-splicing of the ABCC2 gene linked with Bt toxin resistance in Helicoverpa armigera. Sci. Rep..

[11] Zhang S., Cheng H., Gao Y., Wang G., Liang G., Wu K. (2009). Mutation of an aminopeptidase N gene is associated with Helicoverpa armigera resistance to Bacillus thuringiensis Cry1Ac toxin. Insect Biochem. Mol. Biol..

[12] Jin T., Chang X., Gatehouse A.M.R., Wang Z., Edwards M.G., He K. (2014). Downregulation and Mutation of a Cadherin Gene Associated with Cry1Ac Resistance in the Asian Corn Borer, Ostrinia furnacalis (Guenée). Toxins.

[13] Guo Z., Kang S., Chen D., Wu Q., Wang S., Xie W., Zhu X., Baxter S.W., Zhou X., Jurat-Fuentes J.L. (2015). MAPK Signaling Pathway Alters Expression of Midgut ALP and ABCC Genes and Causes Resistance to Bacillus thuringiensis Cry1Ac Toxin in Diamondback Moth. PLoS Genet..

[14] Boaventura D., Ulrich J., Lueke B., Bolzan A., Okuma D., Gutbrod O., Geibel S., Zeng Q., Dourado P.M., Martinelli S. (2020). Molecular characterization of Cry1F resistance in fall armyworm, Spodoptera frugiperda from Brazil. Insect Biochem. Mol. Biol..

[15] Flagel L., Lee Y.W., Wanjugi H., Swarup S., Brown A., Wang J., Kraft E., Greenplate J., Simmons J., Adams N. (2018). Mutational disruption of the ABCC2 gene in fall armyworm, Spodoptera frugiperda, confers resistance to the Cry1Fa and Cry1A.105 insecticidal proteins. Sci. Rep..

[16] Pfaffl M.W., Tichopad A., Prgomet C., Neuvians T.P. (2004). Determination of stable housekeeping genes, differentially regulated target genes and sample integrity: BestKeeper—Excel-based tool using pair-wise correlations. Biotechnol. Lett..

[17] Andersen C.L., Jensen J.L., Ørntoft T.F. (2004). Normalization of Real-Time Quantitative Reverse Transcription-PCR Data: A Model-Based Variance Estimation Approach to Identify Genes Suited for Normalization, Applied to Bladder and Colon Cancer Data Sets. Cancer Res..

[18] Panizzi A.R. (2013). History and Contemporary Perspectives of the Integrated Pest Management of Soybean in Brazil. Neotrop. Èntomol..

[19] Funichello M., Fern J., Grigolli o.J., de Souza B.H.S., Boiccedil A.L., Junior A., Busoli A.C. (2013). Effect of transgenic and non-transgenic cotton cultivars on the development and survival of Pseudoplusia includens (Walker) (Lepidoptera: Noctu-idae). AJAR.

[20] Tabashnik B.E., Gould F., Carriere Y. (2004). Delaying evolution of insect resistance to transgenic crops by decreasing dominance and heritability. J. Evol. Biol..

[21] Marques L.H., Castro B.A., Rossetto J., Silva O.A.B.N., Moscardini V.F., Zobiole L.H.S., Santos A.C., Valverde-Garcia P., Babcock J.M., Rule D.M. (2016). Efficacy of Soybean’s Event DAS-81419-2 Expressing Cry1F and Cry1Ac to Manage Key Tropical Lepidopteran Pests Under Field Conditions in Brazil. J. Econ. Èntomol..

[22] Tindall K.V., Siebert M.W., Leonard B.R., All J., Haile F.J. (2009). Efficacy of Cry1Ac:Cry1F proteins in cotton leaf tissue against fall armyworm, beet armyworm, and soybean looper (Lepidoptera: Noctuidae). J. Econ. Èntomol..

[23] Bel Y., Sheets J.J., Tan S.Y., Narva K.E., Escriche B. (2017). Toxicity and Binding Studies of Bacillus thuringiensis Cry1Ac, Cry1F, Cry1C, and Cry2A Proteins in the Soybean Pests Anticarsia gemmatalis and Chrysodeixis (Pseudoplusia) includens. Appl. Environ. Microbiol..

[24] Jurat-Fuentes J.L., Heckel D.G., Ferré J. (2021). Mechanisms of Resistance to Insecticidal Proteins from Bacillus thuringiensis. Annu. Rev. Entomol..

[25] Bustin S.A., Benes V., Garson J.A., Hellemans J., Huggett J., Kubista M., Mueller R., Nolan T., Pfaffl M.W., Shipley G.L. (2009). The MIQE Guidelines: Minimum Information for Publication of Quantitative Real-Time PCR Experiments. Clin. Chem..

[26] Vandesompele J., De Preter K., Pattyn F., Poppe B., Van Roy N., De Paepe A., Speleman F. (2002). Accurate normalization of real-time quantitative RT-PCR data by geometric averaging of multiple internal control genes. Genome Biol..

[27] Perini C.R., Tabuloc C.A., Chiu J.C., Zalom F.G., Stacke R.F., Bernardi O., Nelson D.R., Guedes J.C. (2021). Transcriptome Analysis of Pyrethroid-Resistant Chrysodeixis includens (Lepidoptera: Noctuidae) Reveals Overexpression of Metabolic Detoxification Genes. J. Econ. Èntomol..

[28] Coates B.S., Siegfried B.D. (2015). Linkage of an ABCC transporter to a single QTL that controls Ostrinia nubilalis larval resistance to the Bacillus thuringiensis Cry1Fa toxin. Insect Biochem. Mol. Biol..

[29] Caccia S., Moar W.J., Chandrashekhar J., Oppert C., Anilkumar K.J., Jurat-Fuentes J.L., Ferré J. (2012). Association of Cry1Ac Toxin Resistance in Helicoverpa zea (Boddie) with Increased Alkaline Phosphatase Levels in the Midgut Lumen. Appl. Environ. Microbiol..

[30] Pigott C.R., Ellar D.J. (2007). Role of Receptors in Bacillus thuringiensis Crystal Toxin Activity. Microbiol. Mol. Biol. Rev..

