# Supplementary material for: Evaluation of Reference Genes and Expression Level of Genes Potentially Involved in the Mode of Action of Cry1Ac and Cry1F in a Susceptible Reference Strain of Chrysodeixis includens
Source: Insects. 2021 Jun 30;12(7):598. doi: 10.3390/insects12070598 (PMC8304518; doi:10.3390/insects12070598)
Supplement: Supplementary file 1 [file insects-12-00598-s001.zip › insects-1265969-supplementary.pdf]

Table. S1 Gene names of orthologous candidate reference genes (*RPL10, GADPH, EF1,* and *ACT1*), potential orthologous Cry toxin target genes, species, GenBank accession numbers and primer sequences for the amplification of the respective *Chrysodeixis includens* genes.

**Gene name (abbreviation)**	**Species**	**GenBank accession number**	**Primer sequence**	**Amplification efficiency % (E)**	**Correlation coefficient (R^2)**
Ribosomal Protein L10 (RPL10)	*Spodoptera exigua*	JF728812.1	5' TCTGCGTCGTGCCAAGTT 3'	101.41	0.997
	Chrysodeixis includens	MT879133	5' AGGGCGGTACTTGACGTTG 3'		
Glyceraldehyde-3-phosphate	*Spodoptera litura*	DQ192234.1	5' ACTACACCGAGGAGCAGGT 3'	101.98	0.996
dehydrogenase (GAPDH)	*Chrysodeixis includens*	MT879136	5' AGCCGAACTCGTTGTCGT 3'		
Elongation Factor 1 (EF1)	*Spodoptera litura*	HQ012003.2	5' TTGCCTGCAAATTCGCCG 3'	101.88	0.998
	Chrysodeixis includens	MT879131	5' ACGCACAGAGGCTTGGAA 3'		
β-Actin 1 (ACT1)	*Spodoptera litura*	KC866373.1	5' TTGCCGGCAACAAGGAAC 3'	109.14	0.996
	Chrysodeixis includens	MT879129	5' CGGTGGCGTCAAATGCTT 3'		
ATP-binding cassette subfamily C2	*Spodoptera frugiperda*	AUO38740.1	5' AGGACCGGTTCTTGTGGA 3'	95.71	0.974
transporter (ABCC2)	*Chrysodeixis includens*	MT879134	5' AACGGGCGCTGGAAAGT 3'		
ATP-binding cassette subfamily C3	*Spodoptera frugiperda*	AUO38741.1	5' TGCCGCTTCCAACCAACT 3'	101.11	0.994
transporter (ABCC3)	*Chrysodeixis includens*	MT879135	5' AGGGCTGCATCAACGGT 3'		
Membrane-bound alkaline	*Lymantria dispar*	AAD31183.1	5' GCCGCCAAAATCGTTGCA 3'	109.70	0.998
phosphatase (mALP)	*Chrysodeixis includens*	MT879132	5' TGGGCTTCTCCTGGAGT 3'		
Aminopeptidase N1 (APN1)	*Plutella xylostella*	AAS75551.1	5' TGTGCTCCTCGTTCACGT 3'	103.00	0.997
	Chrysodeixis includens	MT879125	5' AGCGCCAGTGGTGAAAGT 3'		
Aminopeptidase N2 (APN2)	*Lymantria dispar*	AAL26894.1	5' TCACGCGATGTTCCCTCA 3'	95.00	0.993
	Chrysodeixis includens	MT879126	5' ATGGTTCCGAAGTGGCCA 3'		
Aminopeptidase N3 (APN3)	*Lymantria dispar*	AAL26895.1	5' AACGTTGGGGCAGTGCT 3'	92.40	0.998
	Chrysodeixis includens	MT879127	5' ATGTGCCACGTGGGGTT 3'		
Aminopeptidase N4 (APN4)	*Plutella xylostella*	AHF20243.2	5' ACGCACCTTGGATCGCT 3'	108.30	0.996
	Chrysodeixis includens	MT879128	5' GCGGTTGACAACAGCTGT 3'		
Cadherin (CAD)	*Helicoverpa armigera*	ACZ06062.1	5' TTTGTTCAGGTGCGTGCG 3'	99.80	0.987
	Chrysodeixis includens	MT879130	5' TCACGAAGTCTTCCGGCA 3'		

Table. S2 Toxicity (LC_{50}-and LC_{95}-values) of Cry1Ac and Cry1F to neonates of soybean looper Benzon strain and the respective goodness of fit calculated for the non-linear regressions used

Toxin	N[1]	LC_{50} (ng cm^{-2})	95% CI[2]	LC_{95} (ng cm^{-2})	95% CI	Slope (±SE[3])	df[4]	R^2	SS[5]	Sy.x[6]
Cry1Ac	288	10.41	7.970 - 13.44	44.56	26.54 - 92.27	2.025 (0.40)	17	0.966	1367	8.967
Cry1F	324	194.7	88.44 - 425.7	4,967	2,534 - 10,605	0.909 (0.10)	22	0.954	1545	8.379

[1] N: Number of individuals tested; [2] CI: confidence interval; [3]SE: standard error; [4]df: degree of freedom; [5]SS: sum of squares; [6]Sy.x: standard deviation of the residuals
